# Histology Verification Demonstrates That Biospectroscopy Analysis of Cervical Cytology Identifies Underlying Disease More Accurately than Conventional Screening: Removing the Confounder of Discordance

**DOI:** 10.1371/journal.pone.0082416

**Published:** 2014-01-03

**Authors:** Ketan Gajjar, Abdullah A. Ahmadzai, George Valasoulis, Júlio Trevisan, Christina Founta, Maria Nasioutziki, Aristotelis Loufopoulos, Maria Kyrgiou, Sofia Melina Stasinou, Petros Karakitsos, Evangelos Paraskevaidis, Bianca Da Gama-Rose, Pierre L. Martin-Hirsch, Francis L. Martin

**Affiliations:** 1 Centre for Biophotonics, Lancaster Environment Centre, Lancaster University, Lancaster, United Kingdom; 2 Department of Obstetrics and Gynaecology, Lancashire Teaching Hospitals NHS Foundation Trust, Preston, United Kingdom; 3 Department of Obstetrics and Gynaecology, University Hospital of Ioannina, Ioannina, Greece; 4 Second Department of Obstetrics and Gynaecology, Aristotle University of Thessaloniki - Hippokration Hospital Thessaloniki, Thessaloniki, Greece; 5 Queen Charlotte’s & Chelsea - Hammersmith Hospital, Imperial Healthcare NHS Trust, London, United Kingdom; 6 Department of Surgery and Cancer, Imperial College, London, United Kingdom; 7 Department of Cytopathology, “Attikon” Hospital, University of Athens, Athens, Greece; University of Campinas, Brazil

## Abstract

**Background:**

Subjective visual assessment of cervical cytology is flawed, and this can manifest itself by inter- and intra-observer variability resulting ultimately in the degree of discordance in the grading categorisation of samples in screening *vs.* representative histology. Biospectroscopy methods have been suggested as sensor-based tools that can deliver objective assessments of cytology. However, studies to date have been apparently flawed by a corresponding lack of diagnostic efficiency when samples have previously been classed using cytology screening. This raises the question as to whether categorisation of cervical cytology based on imperfect conventional screening reduces the diagnostic accuracy of biospectroscopy approaches; are these latter methods more accurate and diagnose underlying disease? The purpose of this study was to compare the objective accuracy of infrared (IR) spectroscopy of cervical cytology samples using conventional cytology *vs.* histology-based categorisation.

**Methods:**

Within a typical clinical setting, a total of *n* = 322 liquid-based cytology samples were collected immediately before biopsy. Of these, it was possible to acquire subsequent histology for *n* = 154. Cytology samples were categorised according to conventional screening methods and subsequently interrogated employing attenuated total reflection Fourier-transform IR (ATR-FTIR) spectroscopy. IR spectra were pre-processed and analysed using linear discriminant analysis. Dunn’s test was applied to identify the differences in spectra. Within the diagnostic categories, histology allowed us to determine the comparative efficiency of conventional screening *vs.* biospectroscopy to correctly identify either true atypia or underlying disease.

**Results:**

Conventional cytology-based screening results in poor sensitivity and specificity. IR spectra derived from cervical cytology do not appear to discriminate in a diagnostic fashion when categories were based on conventional screening. Scores plots of IR spectra exhibit marked crossover of spectral points between different cytological categories. Although, significant differences between spectral bands in different categories are noted, crossover samples point to the potential for poor specificity and hampers the development of biospectroscopy as a diagnostic tool. However, when histology-based categories are used to conduct analyses, the scores plot of IR spectra exhibit markedly better segregation.

**Conclusions:**

Histology demonstrates that ATR-FTIR spectroscopy of liquid-based cytology identifies the presence of underlying atypia or disease missed in conventional cytology screening. This study points to an urgent need for a future biospectroscopy study where categories are based on such histology. It will allow for the validation of this approach as a screening tool.

## Introduction

### Lack of Sensitivity and Specificity in Cytology-based Screening

While systematic cervical cancer screening programmes have been shown to reduce disease burden, there remains concern over the lack of sensitivity and specificity with conventional cytological testing. Cytological testing involves collection of exfoliated cells from the cervix and subjective microscopic examination of these cells post-staining. The sensitivity of conventional cytology ranges from 30%–87% [1]–[4]. Several large meta-analyses indicate that both the sensitivity and specificity of cervical cytology is lower than previously estimated [1]–[3]. The low sensitivity for detection of atypia or underlying disease is attributable to sampling and/or laboratory errors but most importantly due to inter-observer error as a result of the subjective nature of cytological interpretation [5]. In spite of the excellent quality of cytology in England, out of 3,759 cytology slides originally classed as normal in patients who subsequently had cervical cancer, only 45% were found to be normal on a review while 11% were inadequate, 21% low-grade (borderline or mild) and 23% high-grade [6]. This was true even for the best laboratories with high quality assurance and fully-trained cytotechnologists. Thus, visual cytology screening has certain inherent limitations.

### Spectroscopy-based Methods as a Novel Screening/Diagnostic Tool

To overcome diagnostic shortcomings in cervical screening, an objective test that can detect dysplasia with a higher sensitivity and also predict irreversible commitment to developing invasive disease is needed. Newer diagnostic tools to improve accuracy and reduce subjectivity are emerging with applicability in both developed and developing countries. Such tools include vibrational spectroscopy [infrared (IR) or Raman], laser-induced fluorescent spectroscopy, optical coherence tomography and confocal imaging. Spectroscopy measurements are derived following the absorption, emission or scattering of electromagnetic radiation by atoms or molecules. The application of various spectroscopic methods to study different forms of biological and/or biomedical material can be termed as biospectroscopy [7]. IR spectroscopy is based on the vibration of atoms within a molecule. When a biological sample is interrogated with IR, an absorbance spectrum is generated which measures the fraction of the incident radiation that is absorbed at different frequencies by the sample. The features of an IR spectrum (*i.e.*, number of IR absorption bands, their intensities and their shapes) are directly related to the molecular structure of a compound. Due to its ability to provide information on fundamental structures of biological compounds, the mid-IR wavenumber region 1,800 cm^−1^ to 900 cm^−1^ is known as “biochemical-cell fingerprint” region.

Attenuated total reflection Fourier-transform IR (ATR-FTIR) spectroscopy is a vibrational spectroscopy method that exploits the ability of cellular biomolecules to absorb in the mid-IR region (λ = 2.5–25 µm) through vibrational transitions of chemical bonds [8], [9]. These vibrations include stretching, bending, rocking, and scissoring movements of the atoms in molecules. It permits the detection and measurement of cellular biomarkers including DNA, RNA, lipids, proteins, phosphates and carbohydrates [8], [10]–[12]. The cellular biomolecules that absorb at different wavenumbers include Amide I (≈1,650 cm^−1^), Amide II (≈1,550 cm^−1^), protein (≈1,425 cm^−1^), Amide III (≈1,260 cm^−1^), asymmetric phosphate stretching vibrations (ν_as_PO_2_^−^, ≈1,225 cm^−1^), carbohydrate (≈1,155 cm^−1^), symmetric phosphate stretching vibrations (ν_s_PO_2_^−^, ≈1,080 cm^−1^) and protein phosphorylation (≈970 cm^−1^).

Raman spectroscopy can be complimentary to IR due to differences in the excitation conditions for both the techniques: change of dipole moment (vector quantity) in the case of an IR spectrum and change of polarization (tensor quantity) in the case of a Raman spectrum. In Raman, the excited vibrational state is directly approached. Raman peaks can collectively provide a ‘biochemical-cell fingerprint’ of the sample, which contains a high profile spectrum of chemical bonds associated with lipids, DNA, RNA and proteins [13]. Mahadevan-Jansen and colleagues reported on the potential use of near-IR Raman spectroscopy to distinguish cervical cytology grades [14]. They developed a compact fibre-optic probe to measure the *in-vivo* Raman spectra of cervical tissue [15]. Further studies have shown promising results for application of this method in grading cervical pre-cancer [16]. In a recent study, confocal Raman spectroscopy coupled with principal component analysis-linear discriminant analysis (PCA-LDA) modelling yielded a diagnostic accuracy of 84.1% (a sensitivity of 81.0% and a specificity of 87.1%) for *in-vivo* discrimination of dysplastic cervix [17]. Apart from being non-destructive and non-invasive, the other distinct advantage of Raman spectroscopy is that it can be used *in vivo,* and is unaffected by water. The limitations are its relatively low sensitivity and undesired fluorescence compared to IR. Relatively expensive and sophisticated instrumentation also should be taken into account.

The Lu*Viva* multimodal hyperspectroscopy (MHS) device has been investigated as a primary screening tool for moderate and high-grade cervical cytology. It detects changes in cervical cells by shining light on the cervix and measuring the patterns of reflected light. Optical coherence tomography (Niris Imaging System), the Epitheliometer, and the Dynamic Spectral Imaging System (DySIS) are other new methods under evaluation, all of which can be used as an adjunct to colposcopy.

### Biospectroscopy as a Novel Screening/Diagnostic Tool

While cytology testing relies on morphological parameters and staining patterns, biospectroscopy records the spectral information from cytology reflecting its biochemical composition, including cells from an interrogated liquid-based cytology (LBC) specimen. Biospectroscopy approaches with multivariate analyses have shown potential to detect cell abnormalities at molecular levels, which occur before the changes in morphology are seen under the light microscope [8], [9], [18], [19]. Spectra obtained with ATR-FTIR spectroscopy differ from normal cytology to atypia and invasive disease [9], [18]–[23]. ATR-FTIR spectroscopy is also capable of distinguishing between hrHPV infections in women exhibiting pre-cancerous cervical cellular atypia [24]. In ATR-FTIR spectroscopy, the signal-to-noise ratio is minimized by setting the spectral acquisition conditions at 8 cm^−1^ resolution, 2.2 kHz mirror velocity and 32 co-additions [8]. Pre-processing of spectra minimizes any interferences caused by instrumentation, sample preparation, and spectra acquisitions.

Preparation of a cytology sample before obtaining spectra takes about 30 minutes with current methods of centrifugation; samples are stored in a desiccator. ATR-FTIR spectroscopy can record 10 spectra from a cytology sample within 15 minutes; this can undoubtedly be automated and speeded up. In conventional cytology, the LBC sample is processed to remove any contaminants and slides are prepared automatically. The process would take on an average 15 minutes [8]. On average, a cytotechnologist would screen 12.5 slides per hour and works for eight hours a day. However, adequate training of a cytotechnologist takes about 2 years and a trained pathologist is still required to review any abnormal cytology and make a final diagnosis [25].

### Validation of Biospectroscopy in Clinical Setting: Subsequent Histology as Gold Standard

While earlier biospectroscopy studies were challenged on grounds that the segregation between cytological categories could be due to contaminated samples, more recent studies using LBC have resolved this issue [18], [20], [26]–[29]. Large-scale studies of IR spectroscopy have emerged in recent years and shown promise for this technique to be used as a screening or diagnostic tool for cervical cancer [19], [29], [30]. However, significant overlap between various cytology categories had been observed suggesting a lower accuracy for IR spectroscopy [29], [31]. This has led to the conclusion that biospectroscopy-based categorisation of cervical cytology is apparently flawed in its ability to detect atypia or underlying disease. However, previous IR spectroscopy studies have utilized cytology-based categorisation as a gold standard to determine the accuracy of biospectroscopy in detection of cytological atypia or underlying disease. Considering the fact that cervical cytology-based categorisation has low sensitivity and specificity as well as poor correlation with subsequent histology, using cytology as a gold standard to ascertain the accuracy of biospectroscopy methods is always likely to generate flawed results. The ideal study to assess the accuracy of biospectroscopy in detecting atypia or underlying disease in cervical cytology should be based on comparisons with corresponding histopathological diagnosis on biopsy, which is considered the gold standard for diagnosis of cervical dysplasia and invasive carcinoma.

### Aims and Objectives

This pragmatic study was carried out in a population undergoing colposcopy-based cervical screening in accordance to the European/Greek/UK NHS guidelines with histological biopsies from a large proportion of participants. Our objective was to determine whether biospectroscopy appears flawed based on imperfect conventional cytological screening categorization or if it can diagnose underlying relevant abnormality based on histological biopsies taken immediately after index LBC.

## Materials and Methods

### Participants

Informed written consent to use cervical tissue for research was obtained from women (*i.e.*, study participants) attending clinics for cervical cytology screening between October 2009 and August 2012. On the advice of the Acting Director of the University General Hospital of Ioannina, Institutional Review Board (*i.e.*, Ethics Committee) approval was obtained [protocol, 28/9-7-2009(s.22)]. Similarly, ethics committee approval was obtained from the local research and ethics committee of Hippokration Hospital at University of Thessaloniki [approval number 3715/21-03-2011] for collection of samples at the Second Department of Obstetrics and Gynaecology, Hippokration Hospital, University of Thessaloniki, Greece. Relevant consent documentation was securely archived in said departments with each patient identifier coded so as to retain the anonymity of study participants. A total of *n* = 350 LBC samples were collected with Rovers™ Cervex-brush in a ThinPrep solution (PreservCyt; Cytyc, USA) from *n* = 281 patients.

### Liquid-based Cytology (LBC) Samples

LBC was repeated immediately before colposcopy assessment and this was the index cytology and not the initial primary screening smear, which might have been assessed in several different laboratories. Each cytology sample underwent cytopathological examination by the resident qualified cytopathologists in two respective university hospitals within quality-assured laboratories. Cervical cytology is graded semi-quantitatively depending on the cellular morphology into Negative, atypical squamous cells of undetermined significance (ASCUS), low-grade squamous intraepithelial lesion (LSIL), high-grade squamous intraepithelial lesion (HSIL) and cancer. Specimens exhibiting viral changes without atypia were classed as HPV or koilocytosis. The corresponding Bethesda grades [32] for cytological abnormalities are as follows: ASCUS and HPV-like feature (borderline nuclear abnormality - BNA); LSIL (mild dyskaryosis); and, HSIL (moderate and severe dyskaryosis). Out of the total number of cytology specimens, *n* = 120 were categorised as Negative, *n* = 88 LSIL, *n* = 50 HSIL, *n* = 50 ASCUS, *n* = 12 HPV, and *n* = 2 cancer. Patients who had unsatisfactory cytology, glandular abnormalities, and those with previous laser treatment, large loop excision of transformation zone (LLETZ), cryotherapy, or other cervical procedures within the previous six months were excluded (*n* = 28).

### Histology Samples

**Figure 1:**
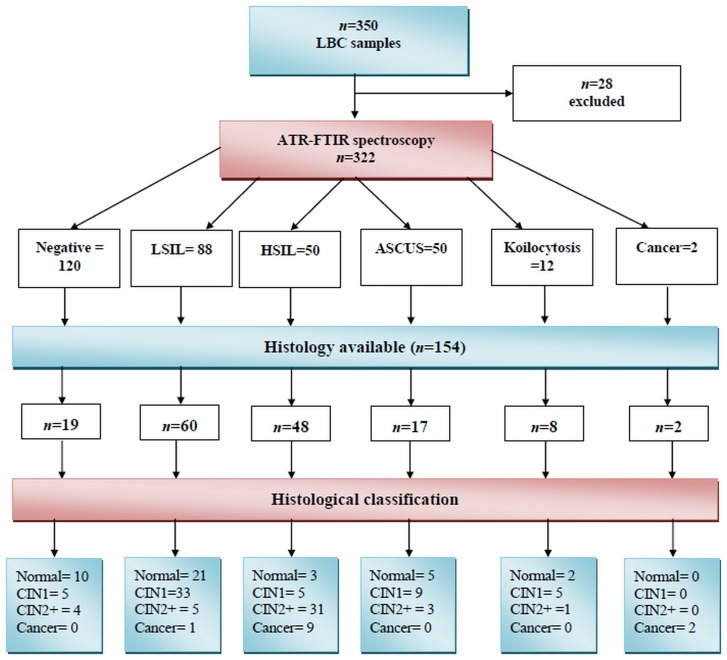
Flow diagram depicting the results of initial cervical cytology (*n* = 322) and subsequent histology (*n* = 154) of liquid-based cytology (LBC) samples. The cytology categories include negative, low-grade squamous intraepithelial lesion (LSIL), high-grade intraepithelial lesion (HSIL), atypical squamous cells of unknown significance (ASCUS), koilocytosis or HPV and cancer. The histological categories include normal, cervical intraepithelial neoplasia grade 1 (CIN1), cervical intraepithelial neoplasia grade 2+ (CIN2+) and cancer.

Patients were managed according to standard protocols in line with European/Greek/UK guidelines. As this was a pragmatic study, colposcopists decided whether to take representative biopsies according to referral smear results, patient characteristics and colposcopic findings. Biopsies were either punch or excisional biopsies. None of these guidelines recommend biopsies after low-grade cytology and negative adequate colposcopy as the only way of minimizing verification bias is by performing loop excision of the transformation, which is associated with long-term fertility morbidity. The histopathological categories for squamous intraepithelial lesion (SIL) are divided into different grades of cervical intraepithelial neoplasia (CIN). Low-grade lesions with less risk of disease development and higher chance of regression are categorised as CIN1 and correspond to LSIL in cytology, while high-grade lesions such as CIN2/CIN3 (also known as CIN2+) exhibit an increased risk of commitment to invasive disease and correspond to HSIL in cytology. The availability of a large proportion of representative follow-up histology for abnormal cytology is the major strength of the longitudinal study, and an overriding weakness of previous spectroscopic studies. Representative cervical tissue for subsequent histology was obtained from almost all the HSIL (48 out of 50; 96%) and the majority of LSIL (60 out of 88; 68%). Biopsy samples were also obtained from a representative proportion of the Negative cytology cohort (19 out of 120; 16%), ASCUS (17 out of 50; 34%) and HPV-like features (8 out of 12; 67%). Fig. 1 shows the flow diagram of cervical cytology classes and follow-up histology results; Table 1 shows the correlation of cytology with subsequent histology. Out of all subsequent histology samples to assess the accuracy of biospectroscopy, *n* = 41 were normal, *n* = 57 CIN1, *n* = 44 CIN2+, and *n* = 12 cancer.

**Table 1:** Correlation of cytology with subsequent histology.

Cytology*	Histology* (*n* = 154)			
	Normal (*n* = 41)	CIN1 (*n* = 57)	CIN2+ (*n* = 44)	Cancer (*n* = 12)
Normal (*n* = 19)	10 (53%)	5 (26%)	4 (21%)	0
LSIL (*n* = 60)	21 (35%)	33 (55%)	5 (8%)	1 (2%)
Koilocytosis or HPV (*n* = 8)	2 (25%)	5 (62%)	1 (13%)	0
HSIL (*n* = 48)	3 (6%)	5 (10%)	31 (65%)	9 (19%)
Cancer (*n* = 2)	0	0	0	2 (100%)
ASCUS (*n* = 17)	5 (30%)	9 (53%)	3 (17%)	0

* Abbreviations: ASCUS, atypical squamous cells of unknown significance, HSIL, high-grade squamous intraepithelial lesion, LSIL, low-grade squamous intraepithelial lesion, CIN, cervical intraepithelial neoplasia, HPV, human papilloma virus.

### Sample Preparation for ATR-FTIR Spectroscopy

**Figure 2:**
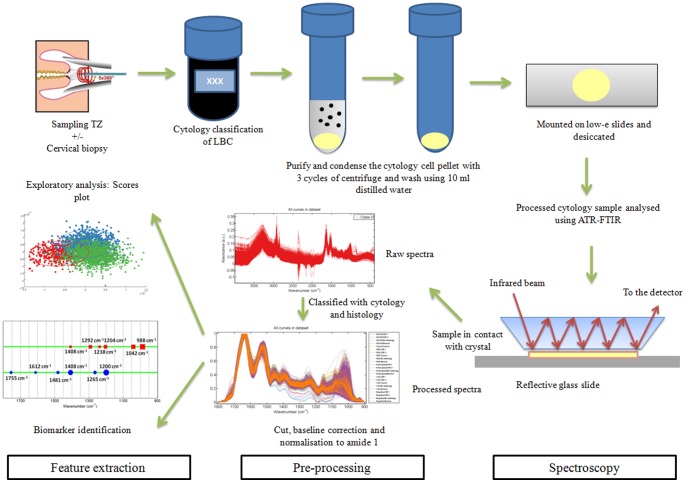
Flow diagram of sample collection, sample preparation, spectroscopy, pre-processing of spectra and feature extraction. After sampling of the transformation zone (TZ), the cells are suspended in ThinPrep solution. An aliquot of 6 ml of cytology specimen was centrifuged at 1,500 rpm for 5 min, after which the supernatant was then aspirated. The remaining cell pellet was re-suspended in 3 ml autoclaved distilled H_2_O and centrifuged at 1500 rpm for 5 min, and the supernatant was again removed. This wash step was repeated three times and, the resulting cell pellet was then re-suspended in 0.5 ml autoclaved distilled H_2_O and transferred to a low-E glass microscope slide. Slides were allowed to air-dry and stored in a desiccator until analysis. Infrared (IR) spectra were obtained using a Bruker TENSOR 27 FTIR spectrometer with Helios ATR attachment containing a diamond crystal. Using a CCTV camera, spectra were acquired from 10 independent sample locations. The datasets were processed using MATLAB R2010a software (Mathworks Inc, Natick, MA, USA) with the IRootLab toolbox (http://irootlab.googlecode.com). IR spectra were pre-processed in three steps, which include cutting, baseline correction and normalization. Feature extraction was carried out using linear discriminant analysis, which allowed for segregation of classes and peak detection plots allowed identification of biomarkers.

An aliquot of 6 ml of cytology specimen in ThinPrep solution was centrifuged at 1500 rpm for 5 min, after which the supernatant (*i.e.*, methanol fixative in ThinPrep) was then aspirated (Fig. 2). Remaining cell pellet was re-suspended in 3 ml autoclaved distilled H_2_O and centrifuged at 1,500 rpm for 5 min, and the supernatant was again removed [8], [9]. This wash step was repeated three times and, the resulting cell pellet was then re-suspended in 0.5 ml autoclaved distilled H_2_O and transferred onto a low-E glass microscope slide (Kevley Technologies, Chesterland, OH, USA). Slides were allowed to air-dry and stored in a desiccator until analysis, thereby removing the possibility of water contributing to the spectral signature.

### Sample Thickness

When transferring the cellular material to a slide, we ensured an even deposition. In ATR-FTIR spectroscopy, an IR evanescent wave penetrates only a few µm into the sample. Samples can therefore be quite thick, with a minimum thickness of 3–5 µm. Too-sparse or too-thin samples will produce spectra of low signal-to-noise ratio. In our studies, we have observed that small areas of thick sample are most appropriate for ATR-FTIR spectroscopy. In addition, we ensured that the sample thickness did not influence the results of the spectra by allowing the Amide I peak of acquired spectra to reach beyond 0.12 a.u. (arbitrary units). This is achieved by adjusting the amount of pressure applied to the region of interest.

### Biospectroscopy: Instrumentation and Parameters

Spectroscopic interrogation of cytology samples was performed with ATR-FTIR spectroscopy at the Centre for Biophotonics (Lancaster University, UK). Cytological and histological diagnoses were unknown to those who performed IR spectroscopy. IR spectra were obtained using a Bruker TENSOR 27 FTIR spectrometer with Helios ATR attachment containing a diamond crystal (Bruker Optics Ltd, Coventry, UK) and operated using OPUS 6.5 software (Bruker Optik GmbH). Using a CCTV camera, spectra were acquired from 10 independent sample locations. The following data acquisition parameters were used: 8 cm^−1^ spectral resolution giving 4 cm^−1^ data spacing, 32 scans, 6 mm aperture setting and 2× Zerofilling factor. The ATR diamond crystal was washed with distilled water and, dried with tissue paper between each sample and before each new slide. A background absorption spectrum (for atmospheric correction) was taken prior to each new sample.

### Spectral Pre-processing

**Figure 3:**
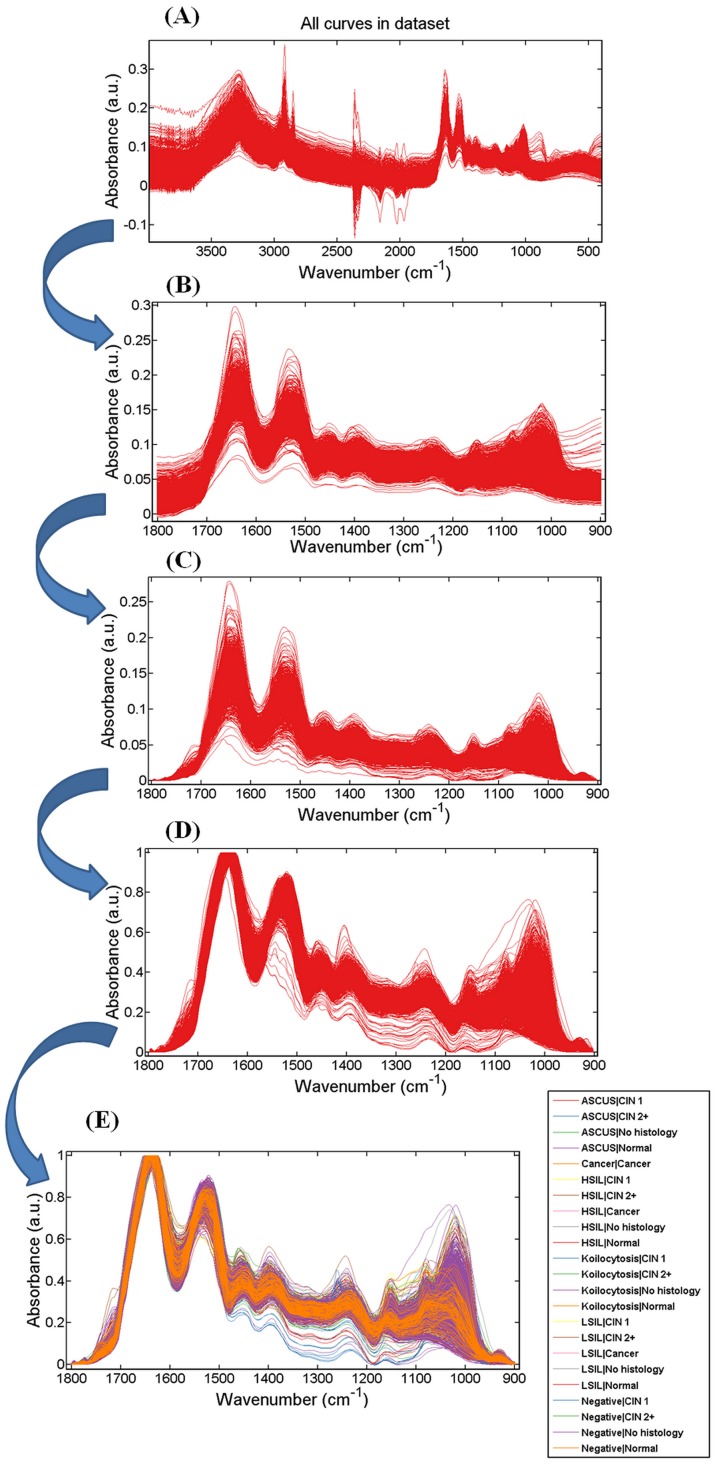
Pre-processing of infrared (IR) spectra obtained from cervical cytology specimens. (**A**) All spectra in the mid-IR region; (**B**) Spectra cut between 1,800 cm^−1^ to 900 cm^−1^; (**C**) Rubberband-like baseline correction; (**D**) Normalization to Amide I peak; and, (**E**) Spectra of *n* = 322 cytology specimens (28 unsatisfactory specimens removed) classed by cytology and histology.

Spectra were converted into absorbance by Bruker OPUS software. The datasets were pre-processed using MATLAB R2010a software (Mathworks Inc, Natick, MA, USA) with the IRootLab toolbox (http://irootlab.googlecode.com; Fig. 3A–3E) [8], [33]. IR spectra were pre-processed in three steps, which include cutting, baseline correction and normalization. This approach is most efficient in maintaining the integrity of the original spectra [8]. Spectra were pre-processed to account and correct for noise, sloping baseline effects, differences in sample thickness/concentration, and to select the regions of interest. In cutting, the spectra are cut between the regions of interest, such as the biochemical-cell fingerprint regions (1,800 cm^−1^–900 cm^−1^), typically containing 235 variables (*i.e.*, 235 wavenumbers at 3.84 cm^−1^ spectral resolution). Baseline correction methods such as “rubberband” aims to correct sloped or oscillatory baselines that arise as a result of physical phenomena such as IR dispersion caused by Mie scattering [34]. Normalization aims to correct for differences in absorbance intensities across spectra that result from variable sample thickness or concentration. Normalization was done by scaling spectra to the highest peak (*i.e.*, Amide I).

### Multivariate Analysis

The data processing is directed towards visualising data as graphs, which allows one to gain insights into underlying biological alterations [35]. Multivariate analysis was performed with LDA using MATLAB (http://www.mathworks.com) with the IRootLab toolbox (http://irootlab.googlecode.com) [8], [33]. Scores plots are used for the visual representation and interpretation of the variables responsible for separation of spectral categories as the Cartesian coordinates after application of linear methods such as principal component analysis (PCA), linear discriminant analysis (LDA) or PCA-LDA. As the numbers of spectra in the cytology dataset (3,220 spectra) are more than five times the numbers of variables (235 data points), we applied LDA as a linear method of spectral analysis [7], [35], [36]. LDA is a supervised technique, which forms linear combinations of variables dependent on differences between the categories in the dataset. It minimises within-category differences (which would mostly be associated with typical heterogeneity) while maximizing between-category discriminating characteristics (*i.e.*, those most likely to be diagnostic).

LDA reduces the numbers of variables within spectral data from 235 to a few (2–7 LDs depending on number of classes). Using linear discriminant 1 and 2 (LD1 and LD2), which represent almost 99% of data, scores plots were obtained for various cytological categories. This pattern-finding LDA approach is to identify how different cytology categories relate to each other (*e.g.*, which categories are closely clustered or distant from each other) and thereby allows one to understand the underlying biochemical alterations responsible for separation of cytology grades. The ellipse around each class represents a 90% confidence interval region. This confidence ellipse or confidence region is used to indicate the reliability of the spectral category means. The level of confidence indicates the probability with which the confidence region captures the “true” (unknown) category mean [37]. Considering cytology as an imperfect tool, IR spectra were subsequently categorised based on subsequent histology and LDA carried out on the histological classes.

Along with the scores plots, the linear method of feature extraction also identifies the contribution of each wavenumber-variable responsible for generating the factors known as loading vectors. A cluster vector approach produces a set of biomarkers per data category [38]–[40]. To obtain a peak detection plot, a cluster vector is drawn from the centre of a reference category (*i.e.*, the “Negative or Normal cytology” group) to the centre of an abnormal cytology cluster in the vector space spanned by the LDA loadings vectors. Subsequently, a peak detection algorithm is applied to identify the six most prominent peaks from each cluster vector and plotted locations of detected peaks along wavenumber line using marker symbols. Size of marker symbol is proportional to the height of their corresponding peak [10]. Absolute values were used to measure the height of the peaks from cluster vector plots in order include both the negative and positive values. Thus, the peak detection plots show the wavenumbers that are responsible for the differences amongst the categories. All wavenumbers are rounded down to the nearest whole number.

Statistical significance of each linear discriminant (LD) contributing to inter-category segregation for cytology/histology correlation of normal and high grade histology was determined by the Dunn’s multiple comparison test (Prism 5, GraphPad Software Inc., La Jolla, CA, USA). Dunn’s test compares the difference in the sum of ranks between two columns with the expected average difference (based on the number of groups and their size). The performance of two algorithms is significantly different if the corresponding average of rankings is at least as great as its critical difference. It is also referred to as post-test following a Kruskal-Wallis test [41], [42].

## Results

### Linear Discriminant Analysis (LDA) of IR Spectra Based on Conventional Screening

**Figure 4:**
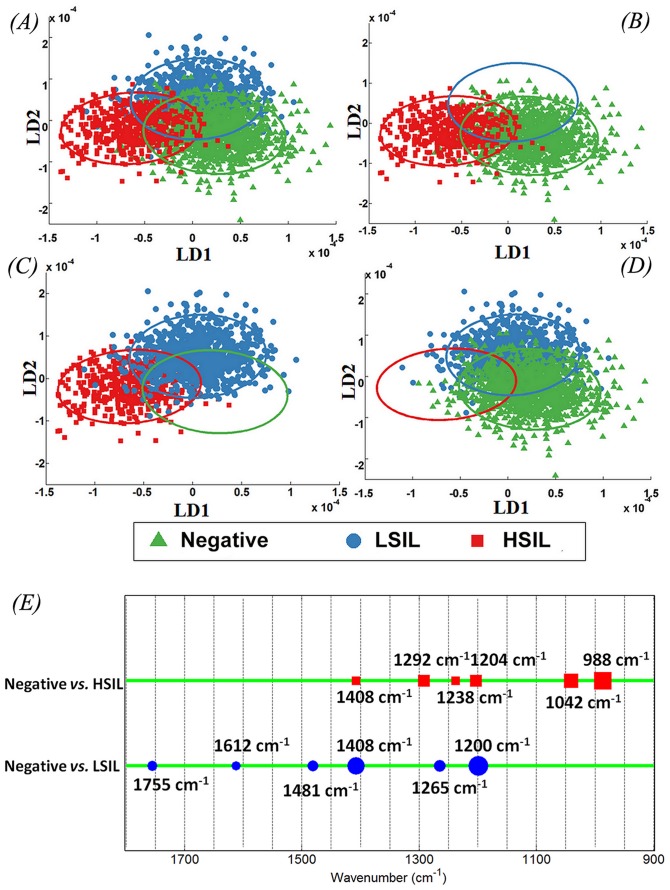
Comparison of infrared (IR) spectra from LBC samples using LDA scores plot with confidence ellipse representing confidence interval at 90% for the observed values. (**A**) Negative *vs.* LSIL *vs.* HSIL; (**B**) Negative vs. HSIL; (**C**) LSIL *vs.* HSIL; and, (**D**) Negative *vs.* LSIL; (**E**) Corresponding peak detection plot with top six wavenumbers responsible for segregation of Negative from LSIL and HSIL.

The variations of IR spectra for Negative (*n* = 120), LSIL (*n* = 88) and HSIL (*n* = 50) were examined. Significant overlap was observed in the LDA-derived scores plots for all three grades with only minimal evidence of segregation between categories (Fig. 4A). The overlap region was smaller for Negative *vs.* HSIL (Fig. 4B) and LSIL *vs.* HSIL (Fig. 4C) as compared to Negative *vs.* LSIL (Fig. 4D). Fig. 4E shows the top six peak assignments responsible for segregation IR spectra of Negative from LSIL and HSIL.

**Figure 5:**
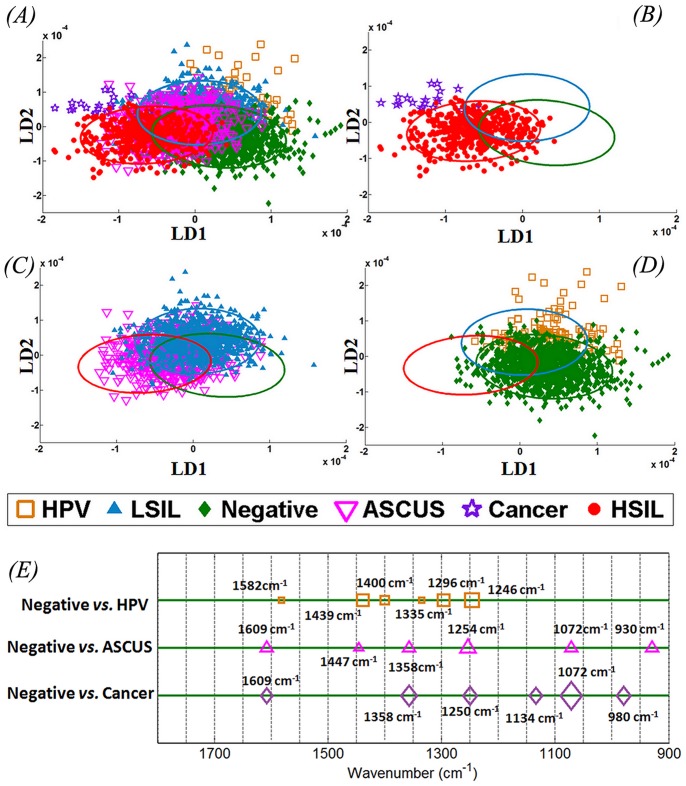
Comparing various cytology grade spectra using multivariate analysis. (**A**) Comparison of spectral points for Negative *vs.* LSIL *vs.* HSIL (closed symbols) with HPV *vs.* ASCUS *vs.* Cancer (open symbols). Scores plots with confidence ellipse showing the relationship of (**B**) cancer with HSIL; (**C**) ASCUS with LSIL; and, (**D**) HPV-like features with Negative cytology; (**E**) Peak detection plot following linear discriminant analysis (LDA) showing top six wavenumbers responsible for segregation of Negative cytology from ASCUS, HPV and Cancer cytology.

Apart from the aforementioned three categories (*i.e.*, Negative, LSIL and HSIL), the IR spectra of ASCUS (*n* = 50), HPV-like features (*n* = 12) and cancer (*n* = 2) were also studied. Fig. 5A shows the 2-D scores plot after LDA of the IR spectra from all six cytological grades exploiting the similarities and differences in their biomolecular alterations as revealed by overlap and separation of spectral points. While the spectral points of cancer on cytology clustered remarkably in close proximity to HSIL (Fig. 5B), the IR spectra of ASCUS showed a distribution of points across all three categories with no specific pattern (Fig. 5C). The HPV-like features on cytology (Fig. 5D) were mostly clustered close to LSIL with some distribution near the Negative cytology. Fig. 5E shows the peak detection plot with the top six wavenumbers that resulted in segregation of Negative from HPV, ASCUS and Cancer spectra.

It is known that the histopathological diagnosis of given grades of CIN has poor reproducibility [43], [44]. Despite this, the separation of CIN into three subcategories (*e.g.*, CIN1, CIN2 and CIN3) correlates to a general extent with rates of development and regression of the intraepithelial lesion [45]. Histology is considered the gold standard for diagnosis of underlying pre-invasive disease in cervical screening. Out of all histology specimens (*n* = 154), nineteen were associated with Negative cytology. The correlation of Negative cytology with concurrent histology was poor with only about half of the patients with Negative cytology showing no histopathological abnormality. Thus, the false negative rate was very high with about 47% of the Negative cytology having CIN on histology. Out of *n* = 40 patients with HSIL, 65% (31/48) had high-grade CIN, 19% (9/48) had cancer, 10% (5/48) had CIN1 and 6% (3/48) had normal histology. When patients (*n* = 68) with low-grade cytology (LSIL/HPV-like features) were classed on subsequent histology; 56% had CIN1, 9% had high-grade CIN, one had cancer and 34% had normal histology. When ASCUS cytology was determined by follow-up histology, about half were CIN1 (9/17), 30% (5/17) were normal and 17% (3/17) had high-grade CIN. In ASCUS, the cytology displays cellular abnormalities more marked than simple reactive changes (atypical squamous cells), but does not meet criteria for squamous intraepithelial neoplasia. Thus, ASCUS is likely to represent the normal or low-grade cytological change; however, the potential to have high-grade disease within this category exists.

### Categorisation of IR Spectra Based on Gold Standard Subsequent Histology

**Figure 6:**
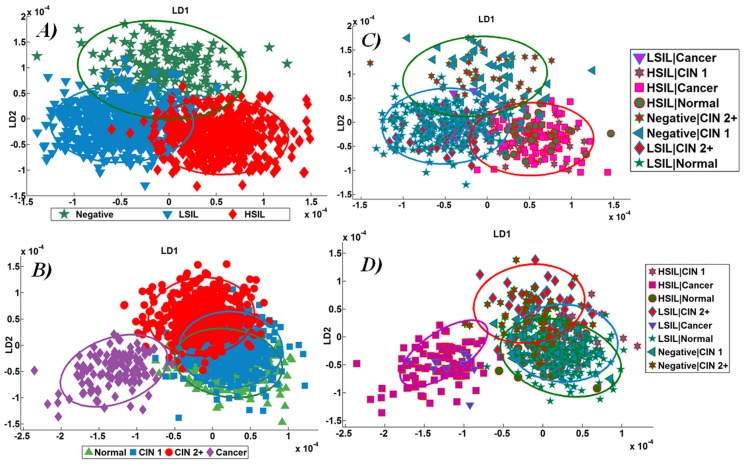
Analysis of infrared (IR) spectra from cervical cytology samples with subsequent histology (*n* = 154). LD1 and LD2 were used to plot the confidence ellipses for cancer (violet), high-grade lesion (red), low-grade lesion (blue) and Negative (green). (**A**) Comparison of linear discriminant analysis (LDA) scores plots of IR spectra according to conventional cytology grades: Negative *vs.* LSIL *vs.* HSIL; (**B**) Comparison of LDA scores plots according to subsequent histology grades: Normal *vs.* CIN1 *vs.* CIN2+ *vs.* Cancer. In the next two figures, the scores plot for discordant cytology and histology were depicted by double-coloured symbols, with the outer colour determining the cytology grade and the inner colour determining the histology grade; (**C**) Comparison of LDA scores plots derived from IR spectra analysed using cytology-based categories; and, (**D**) Comparison of LDA scores plots derived from IR spectra analysed using subsequent histology-based categories.

In this analysis, the IR spectra of LBC specimens with subsequent follow-up histology results available (*n* = 154) were analysed according to cytological and histological categories. Fig. 6A shows a 2-D scores plot after LDA of IR spectra from Negative, LSIL and HSIL cytology as categorised by the cytology and Fig. 6B shows a 2-D scores plot of IR spectra of cytology as classed by corresponding subsequent histology. Fig. 6C and 6D shows the 2-D scores plots with confidence ellipses and double-coloured spectra. The IR spectral points with agreement between cytology and subsequent histology are within the confidence ellipse while the spectral points for the discordant grades of cytology are depicted as double-coloured symbols.

When IR spectra are analysed using cytology grades (Fig. 6A), poor correlation of spectral points with histology is observed (Fig. 6C). This is because the cytology-based grading ignores the discrepancy (discordance) between the cytology and histology. In other words, this can be regarded as a teaching error. The observed overlap of spectral points suggests that perhaps biospectroscopy-based classification of spectral cytology is not very robust. However, when the IR spectra of cytology are graded according to histology (Fig. 6B), the correlation of spectral points with histology is highly accurate (Fig. 6D). The histology-based grading of IR spectra derived from cytology samples are depicted by double-coloured scores in Fig. 6D and explains the overlap regions seen in Fig. 6B. Thus, multivariate analysis of the cytology-derived IR spectra using histology results in accurate assignment of categories irrespective of discordant cytology (see the double-coloured scores plots in Fig. 6C and 6D).

Negative screening and high-grade intraepithelial lesions are the two most common results that influence the risk of developing cervical cancer and therefore the treatment choice. While no intervention would be required in Negative cytology, high-grade lesions require abnormal cells to be removed with surgical intervention. The IR spectra from these two cytological categories were further characterised for accuracy of identification of corresponding histological category.

**Figure 7:**
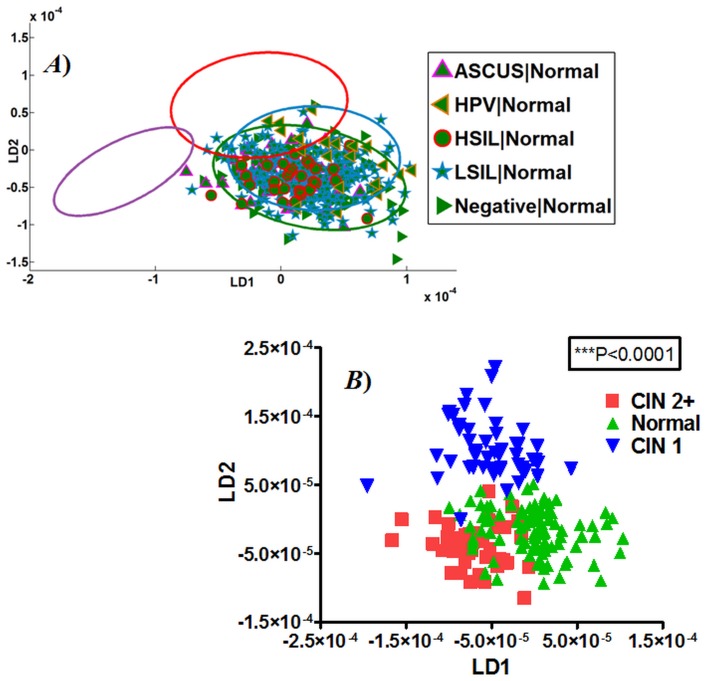
Infrared (IR) spectral points of cytology in relation to normal histology. (**A**) IR spectra for normal histology classified according to the screening cytology results and in relation to the confidence ellipses of histology grades; and, (**B**) Analysis of IR spectra from normal cytology classified according to subsequent histology.

The IR spectra of patients with normal histology but differing cytology grades were plotted in relation to the confidence ellipse for histological categories (Fig. 7A). Despite the differing grades of cytological atypia as determined by imperfect cytological screening, the IR spectral points for normal histology were seen clustering within the ellipse of normal histology in a 2-D scores plot. The spectra for HPV with normal follow-up histology were seen in the overlap region of normal and low-grade histology. Furthermore, when spectral points for Negative cytology were projected as LDA scores plots according to the subsequent histology (Fig. 7B), clear differences are observed (Table 2) between normal *vs.* CIN1 (*P*<0.001) and CIN2+ *vs.* CIN1 (*P*<0.001). The CIN2+ *vs.* Normal spectral categories exhibit good segregation (*P*<0.05) with some overlap, possibly due to the limited number of specimens within the Negative cytology category (*n* = 10).

**Table 2:** Spectral variations amongst histology of Negative cytology.

Dunn’s Multiple Comparison Test	Difference in rank sum	*P*-value	Summary
CIN2+ *vs.* Normal	−29.47	*P*<0.05	*
CIN2+ *vs.* CIN1	−114.9	*P*<0.001	***
Normal *vs.* CIN1	−85.44	*P*<0.001	***

**Figure 8:**
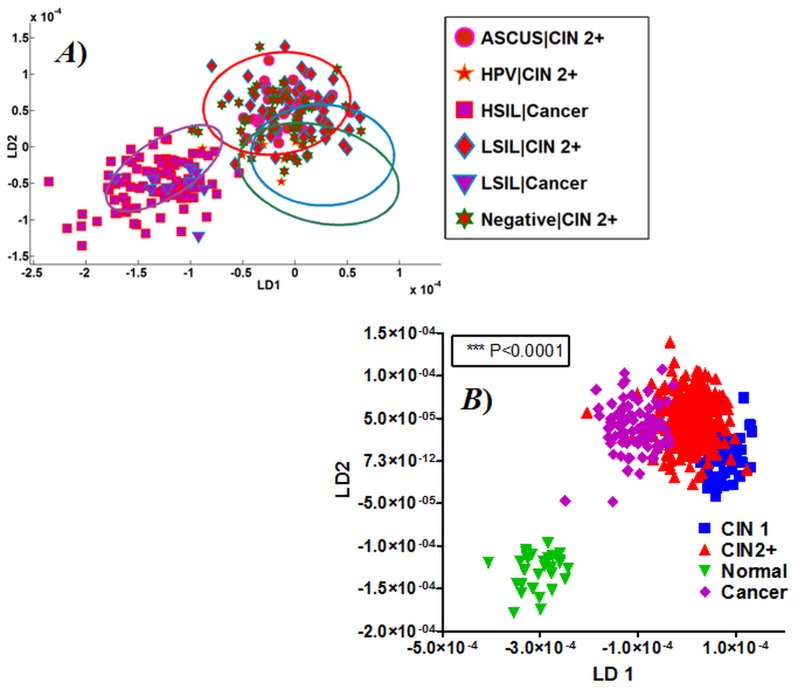
Infrared (IR) spectral points of cytology in relation to high-grade histology. (**A**) IR spectra for high-grade histology (CIN2+ and cancer) classified according to the screening cytology result and in relation to the confidence ellipses of histology grades; and, (**B**) Analysis of IR spectra of high-grade lesions cytology classified according to subsequent histology.

The cytological spectra of LBC specimens with histology suggestive of CIN2+ and cancer were analysed in relation to the initial screening cytology. A remarkable degree of agreement was observed in the spectral points of IR spectra for high-grade lesions on histology, irrespective of the varying degree of discordance in initial cytology (Fig. 8A). The spectral points of Negative cytology but high-grade histology are seen in HSIL ellipse. As seen in Fig. 8A, ASCUS with CIN2+ histology clustered within the high-grade lesion ellipse. The spectral points for CIN2+ in HPV cytology clustered in the overlap region between all three categories but more towards the high-grade ellipse (Fig. 8A). When spectra for HSIL were classed on LDA using histology (Fig. 8B), excellent separation of normal from all other grades was observed (Table 3). It is worth mentioning that the cancer category for cytology and histology are of a limited sample size (*n* = 12 and 2, respectively), which is likely to influence the analysis.

**Table 3:** Spectral variations amongst histology of HSIL cytology.

Dunn’s Multiple Comparison Test	Difference in rank sum	*P*-value	Summary
CIN1 *vs.* CIN2+	−156.2	*P*<0.001	***
CIN1 *vs.* Normal	107.8	*P*<0.01	**
CIN1 *vs.* Cancer	−123	*P*<0.001	***
CIN2+ *vs.* Normal	264	*P*<0.001	***
CIN2+ *vs.* Cancer	33.18	*P*>0.05	ns*
Normal *vs.* Cancer	−230.8	*P*<0.001	***

* Abbreviation: ns, not significant.

One of the major advantages of biospectroscopy over other analytical methods is the ability to identify most important discriminating wavenumber-variables to lend further insights into biological mechanisms. The responsible wavenumbers with their biochemical assignments for segregation of Negative cytology from LSIL were mostly attributed to DNA/RNA ν_as_PO_2_^−^ (1,200 cm^−1^) and COO- symmetric stretching vibrations of fatty acids and amino acid (1,408 cm^−1^) (Fig. 4E; Table 4). Similar wavenumber contributions have been reported for inter-category variance between Negative *vs.* LSIL [9], [19]. The wavenumbers segregating Negative from HSIL are assigned to protein phosphorylation (988 cm^−1^) and glycogen (1,042 cm^−1^) alterations; in contrast, LSIL *vs.* HSIL were segregated due to alterations in proteins (1,481 cm^−1^) and protein phosphorylation (988 cm^−1^). Glycogen, which is abundant in normal squamous epithelial cells of the cervix, contributes significantly to the segregation of normal cells from high-grade lesions. However, the increasing grades of squamous neoplasia from LSIL to HSIL result in alterations mainly in intracellular proteins and membrane proteins/lipids. The important wavenumber assignments for Negative *vs.* HPV were mainly attributed to Amide III (1,246 cm^−1^) and protein vibrations (1,439 cm^−1^) while those for Negative *vs.* ASCUS were due to protein phosphorylation (930 cm^−1^) and Amide III (1,254 cm^−1^). The wavenumber assignments separating Negative from cancer were ν_s_PO_2_^−^ (1,072 cm^−1^) and COO- symmetric stretching vibrations of fatty acids and amino acid (1,358 cm^−1^) (Fig. 5E; Table 5).

**Table 4:** Top six wavenumbers responsible for separating negative, LSIL and HSIL.

Comparisons	Wavenumbers	Tentative assignments
Negative *vs*. HSIL	988 cm^−1^	Protein phosphorylation
	1042 cm^−1^	Glycogen
	1204 cm^−1^	C-O, C-N, C-C bonds
	1238 cm^−1^	Asymmetric phosphate stretching vibrations (ν_as_PO_2_^−^)
	1292 cm^−1^	Amide III
	1408 cm^−1^	COO- symmetric stretching vibrations of fatty acids and amino acid
Negative *vs*. LSIL	1200 cm^−1^	C-O, C-N, C-C bonds of DNA/RNA ν_as_PO_2_^−^
	1265 cm^−1^	Amide III
	1408 cm^−1^	COO- symmetric stretching vibrations of fatty acids and amino acid
	1481 cm^−1^	Proteins?
	1612 cm^−1^	Amide I
	1755 cm^−1^	Lipids
LSIL *vs*. HSIL	988 cm^−1^	Protein phosphorylation
	1188 cm^−1^	Carbohydrate
	1292 cm^−1^	Amide III
	1481 cm^−1^	Proteins
	1620 cm^−1^	Amide I
	1755 cm^−1^	Lipids

**Table 5:** Top six wavenumbers responsible for separating negative cytology from HPV, ASCUS and Cancer.

Comparisons	Wavenumbers	Tentative assignments
Negative Vs HPV	1246 cm^−1^	Amide III
	1296 cm^−1^	Amide III
	1335 cm^−1^	COO- symmetric stretching vibrations of fatty acids and amino acid
	1400 cm^−1^	COO- symmetric stretching vibrations of fatty acids and amino acid
	1439 cm^−1^	Proteins
	1582 cm^−1^	Amide II
Negative Vs ASCUS	930 cm^−1^	Protein phosphorylation
	1072 cm^−1^	Symmetric phosphate stretching vibrations (ν_s_PO_2_^−^)
	1254 cm^−1^	Amide III
	1358 cm^−1^	COO- symmetric stretching vibrations of fatty acids and amino acid
	1447 cm^−1^	Proteins?
	1609 cm^−1^	Amide I
Negative Vs Cancer	980 cm^−1^	Protein phosphorylation
	1072 cm^−1^	ν_s_PO_2_^−^
	1134 cm^−1^	C-O stretch (nu CO)
	1250 cm^−1^	Amide III
	1358 cm^−1^	COO- symmetric stretching vibrations of fatty acids and amino acid
	1609 cm^−1^	Amide I

## Discussion

The objective of cervical cancer screening programmes is to reduce the incidence and mortality from this disease by identifying women with pre-cancerous cervical lesions and early invasive cancers, and treating these individuals appropriately. Establishing national cervical cancer screening programmes in developing countries has failed as a result of the complexity of the infrastructure required and the intensity of competing health needs [23]. Part of the problem in running a successful programme is the poor sensitivity and a poor positive predictive value of cervical cytology [4], [46], [47]. High-risk HPV-DNA testing has higher sensitivity but lower specificity than cytology [48]. Visual inspection with acetic acid (VIA) is a low-cost, point-of-care test but suffers from a poor positive predictive value with the risk of significant over-treatment [49]. The search for newer low-cost technologies in cervical cancer screening thus continues. To use inexpensive technology such as biospectroscopy as a novel screening tool requires thorough evaluation and assessment. A part of the process of technology assessment for a novel diagnostic tool is to identify the appropriate gold standard.

Previous studies determining accuracy of biospectroscopy have used cytology as a gold standard. When an imperfect gold standard is used to determine disease status, significant bias can be introduced into measures of a test’s performance. Histology should be used as a gold standard to determine the accuracy of a new screening test for pre-invasive cervical disease.

The accuracy of biospectroscopy of LBC samples as a novel screening test was compared with conventional cytology screening using histology as a gold standard. While, conventional cytology showed a very poor correlation with histology, IR spectra from LBC specimens showed a more robust segregation of categories when subsequent histology was used as a gold standard for analysis. Additionally, the IR spectra of ASCUS and HPV-like features on cytology appeared to segregate according to their subsequent histology grades. Thus, biospectroscopy identifies underlying pre-invasive cervical disease more accurately than conventional cytology when their performance is determined by subsequent histology.

Over the last two decades, many researchers across the globe have applied FTIR spectroscopy to detect pre-invasive disease in cervical cytology [8], [9], [18], [22], [28]. Studies employing multivariate classification analysis have shown good segregation of cytology grades [18]. However, most studies determine IR spectroscopy to be an imperfect tool for assessment of cervical cytology [11], [28]. A recent study was unable to show a significant improvement in class segregation of cytology grades despite using a large number of LBC specimens to assess the accuracy of IR spectroscopy [29]. Clearly, the inherent weakness within these studies was the use of cervical cytology (an imperfect gold standard) grades to compare the performance accuracy of IR spectroscopy with multivariate analysis.

In the current study, we prospectively collected histology from patients undergoing cervical cytology and used it as a gold standard for the analysis of IR spectra from cytology specimens. Initial multivariate analysis of IR spectra using cytological categories showed imperfect categorisation with significant overlap based on conventional cytology screening grades (Figs. 4 and 5). However, when IR spectra were classed using subsequent histology, the spectral points showed discrimination of cytology grades in accordance with the pattern of underlying disease (Fig. 6). IR spectra from cytological categories at the beginning and the end of the disease spectrum (Negative and HSIL) were further scrutinised based on histology (Figs. 7 and 8). These analyses explained the segregation of scores plots in the overlap region (Figs. 7B and 8B).

When IR spectra were categorised by histology grades, high-grade lesions (CIN2+ and cancer) segregated from normal and low-grade lesions. However, clear overlap in the spectral points of low-grade lesion and normal is observed (Fig. 6B & D). Interestingly, when classed by histology (Fig. 6D), the spectral points of IR spectra in the overlap region were of the discordant cytology category (cytology not in agreement with histology). Thus, biospectroscopy of cytology spectra appears imperfect due to the discordance between cytology and histology. Histology reflects the actual underlying disease status. Therefore, the accuracy of IR spectra of cytology is best scrutinized based on the histological categories.

The IR spectral-discordant categories for Negative and HSIL cytology were further characterized based on dual categorisation with cytology and histology. In conventional cytology, 47% of the Negative cytology had CIN1 and about 10% of LSIL had CIN2+/cancer on histology. Thus, cytological screening fails to detect the true underlying disease in a proportion of LBC specimens resulting into a significant under-grading of abnormalities. When biospectroscopy analysis was applied to these LBC specimens, the spectral points for discordant grades (Negative cytology but CIN on histology) of IR spectra showed excellent segregation (Fig. 7B; Table 2). A retrospective review suggested that a high proportion of Negative cytology taken up to three and half years before diagnosis of cancer had abnormal cells [6]. Biospectroscopy has the potential to identify these “missing” abnormal cells in the apparently Negative cytology specimen.

Identification of benign lesions (normal) in high-grade cytology has a potential to prevent unnecessary surgical excision biopsies or repeat cytology testing. Out of 48 patients with HSIL, 16% were normal or CIN 1 on histology. The IR spectra of LBC specimens with normal histology segregated extremely well from low-grade and high-grade lesions (Fig. 8B). Additionally, biospectroscopy was able to segregate the IR spectra of ASCUS, low-grade and HPV diagnosis in cytology but with a high-grade histology. ASCUS with high-grade histology (ASCUS/CIN2+) could reflect atypical squamous cells: high-grade (ASC-H), which carries a higher risk of progression to high-grade lesion and if it remains undiagnosed may lead to the development of cervical cancer. Biospectroscopy may identify ASC-H with high likelihood of disease progression amongst the ASCUS cytology.

This study has some limitations. We did not have histology for all cytological categories. Therefore, a possibility of selection bias towards high-grade lesions cannot be ruled out. We have used histology as a gold standard while the possibility of inter-observer variability in histological classification of low-grade lesions cannot also be completely eliminated. The only way to minimize this verification bias is to perform a LLETZ cervical procedure, which provides a sample for histological verification. However, in women with low-grade cytological abnormalities and negative colposcopy, the consensus view is that this is unethical as the short and long-term sequalae of LLETZ outweigh the minimal risk of high-grade disease being present. Despite these limitations, it is clear from the current study that biospectroscopy has the potential to identify underlying cervical atypia or invasive disease. Use of cytology-based categorisation of IR spectra leads to poor assignment of categories resulting into significant overlap in spectral points. When histopathological categories are used to carry out multivariate analysis, biospectroscopy is able to determine the atypia or invasive disease more accurately. Within histology-based categories, some overlap was observed between classes although the segregation was better than cytological-based categorisation. Although, histological diagnosis is considered gold standard, its limitations are well documented [6], [43], [50]. Similar to cytology, histological diagnosis is dependent on interpretation by one or more pathologists and is subjected to inter-observer variability. Another possibility is that the biopsy is obtained from an area not affected by SIL on the cervix. A US-based multicentre trial has shown that the histopathologic interpretation of excision or a punch biopsy can be extremely variable, even between the pathologists at academic medical institutions [50]. Only 43% of low-grade dysplasia interpretations were reproducible while 90% of normal and 76% of high-grade dysplasia results showed an agreement [50]. Thus, one of the inherent difficulties in assessing the accuracy of any new technology is a comparison with a flawed gold standard.

Overall, the underlying pre-invasive cervical disease is accurately identified with biospectroscopy when the histology rather than conventional screening grades are used as gold standard. Future studies should exercise caution in interpreting and concluding results that are just based on imperfect cytology grades without histology. It is unknown whether the crossover in histological class is a consequence of imperfect histology or evidence of the multi-stage process of transformation towards disease development. To identify this, sequential samples are required over a period of time from the same patient. Biospectroscopy is an inexpensive, automatable tool based on objective spectral criteria that does not require highly-skilled personnel. It caries a potential to either be a screening tool in cervical cytology or can be used as an adjunct to identify discordant cytology in conventional screening, thus eliminating unnecessary referrals and treatments and subsequently reducing anxiety and long-term post-treatment obstetric sequelae [51]–[54]. It is possible to use spectroscopy as an adjunct to cytology applying it to identify the correct categories for false positive and false negative cytology. Such an approach would allow for resolution of cytology-histology discrepancies. The cytology laboratory will have centrifuge therefore the only additional cost would include an ATR-FTIR spectrometer; this is not envisaged as a major obstacle. Future studies should be caution in interpreting and concluding results just based just on imperfect cytology grades without histology.
